# Mining resistance genes of soybean cyst nematode based on genome-wide association study

**DOI:** 10.3389/fpls.2026.1834180

**Published:** 2026-06-11

**Authors:** Haibo Hu, Zhihui Cui, Depeng Wu, Qingyan Zhang, Xiaolei Wang, Yingchun Liu, Zeran Kang, Xue Wei, Liuxi Yi, Yang Wu, Huimin Shi, Yunshan Wei, Xuechao Zhou

**Affiliations:** 1 Chifeng Academy of Agricultural and Animal Husbandry Sciences, Chifeng, Inner Mongolia, China; 2 Collaborative Innovation Center of Regional Modern Agriculture & Environmental Protection Co-constructed by the Province and Ministry, Huaiyin Normal University, Huai’an, China; 3 Chifeng Meteorological Bureau, Chifeng, Inner Mongolia, China; 4 College of Agriculture, Inner Mongolia Agricultural University, Hohhot, Inner Mongolia, China

**Keywords:** candidate gene, genome wide association study, overexpressing plants, single nucleotide polymorphism, soybean cyst nematode

## Abstract

Soybean cyst nematode disease is one of the major threats to global soybean production, characterized by its widespread distribution, severe damage, and strong pathogenicity. Identifying and regulating key resistance genes, as well as incorporating them into cultivated varieties through genetic transformation, represents a relatively efficient and cost-effective prevention and control strategy. From 2022 to 2023,at the Chengzi and Xiqiao experimental bases of the Chifeng Academy of Agriculture and Animal Sciences, this study utilized 206 soybean materials for resistance evaluation under two-year, four-site environmental conditions. Based on 101,549 high-quality single nucleotide polymorphism (SNP) loci derived from sequencing data, phylogenetic analysis and principal component analysis were conducted. Genome-wide association study (GWAS) identified 19 SNP loci significantly associated with resistance to soybean cyst nematode, distributed across 11 chromosomes. Among these, four loci (Chr18_56072377, Chr08_159841, Chr15_50312327, and Chr16_32880873) were detected under multiple environmental conditions, with effect values ranging from -35.75 to 24.25.The genes associated with these loci were enriched in multiple pathways, including cell wall construction, root development, and amino acid metabolism. The disease resistance function of the candidate gene *Glyma.18G279900* was preliminarily validated. This study addresses the gap in identifying stable resistance loci across diverse environments, advances the understanding of resistance-related metabolic regulatory mechanisms, provides new markers and gene resources for disease-resistant molecular breeding, and offers direct application value for cultivating resistant varieties adapted to local ecological conditions.

## 1 Introduction

Soybean Cyst Nematode (SCN) is a significant pathogen severely threatening global soybean production, characterized by a wide host range, strong environmental adaptability, and persistent soil survival, leading to substantial yield losses and compound infections (Tian et al., 2007). Although chemical control methods exhibit some effectiveness, they suffer from environmental pollution and sustainability limitations, making the development and utilization of resistant varieties the most economically efficient and effective control strategy (Chen et al., 2021). Following the completion of the soybean whole-genome sequencing in 2010 (Schmutz et al., 2010) soybean genomics research has advanced rapidly (Chang et al., 2018) Genome-wide association study (GWAS) has become a core technical approach for dissecting the complex genetic mechanisms underlying SCN resistance. Compared to traditional linkage analysis based on biparental hybrid populations, GWAS leverages historical recombination events accumulated in natural populations, enabling higher-resolution genome-wide scanning, particularly suitable for uncovering complex resistance traits controlled by multiple minor-effect genes (Lyu et al., 2017). This method has been successfully applied to the study of various soybean agronomic traits, providing important molecular markers and candidate genes for molecular breeding.

In the study of biotic stress resistance, GWAS technology has also demonstrated significant potential. In resistance studies on rice bacterial blight (Jing et al., 2019) cucumber powdery mildew (Shim et al., 2019) and soybean lodging resistance (Li et al., 2019) multiple resistance loci and candidate genes have been successfully identified. Specifically for SCN resistance research, GWAS enables rapid scanning of SNP markers across the genome to identify loci significantly associated with SCN resistance phenotypes. For example, Jain (Jain et al., 2019) conducted GWAS on 317 accessions from the USDA core collection and identified homologous genes of the soybean rhg1 locus on chromosomes Pv01 and Pv08 in the Mesoamerican and Andean gene pools, which confer resistance to SCN. Similarly, Yu (Yu et al., 2023) performed GWAS on 481 soybean accessions and identified 23 genomic regions associated with resistance to SCN3,further validating the effectiveness of GWAS in locating SCN resistance loci. By analyzing genetically diverse natural populations through GWAS, resistance alleles underutilized in traditional disease resistance breeding can be mined, broadening the genetic basis of SCN resistance in soybeans (Kandoth et al., 2017; Kofsky et al., 2021).

GWAS results help clarify whether SCN resistance is controlled by a few major genes or by the cumulative effects of multiple minor genes, thereby deepening the understanding of its genetic architecture (Pokhare et al., 2020). Based on genomic regions harboring significant association signals, key candidate genes can be efficiently screened by integrating gene annotation, expression profiles, and other data, providing direct targets for subsequent functional validation and breeding applications through transgenic or gene editing approaches (Patil et al., 2019; Atnaf et al., 2021). However, current GWAS studies on SCN resistance still have certain limitations: on the one hand, most studies use relatively small population sizes (typically 120–200 accessions),which may limit the detection capability for rare alleles and statistical power (Li et al., 2022) on the other hand, environmental stability is critical for the application of resistance loci in breeding practice, yet most studies lack multi-environment, Ulti-year phenotypic evaluations, resulting in insufficient assessment of genotype-by-environment interaction effects for the identified loci (Chen et al., 2022). Furthermore, the understanding of the complex molecular regulatory networks underlying SCN resistance, particularly at the metabolic pathway level, remains inadequate.

To address these gaps, this study aims to: (1) systematically evaluate SCN resistance under multi-environment conditions (two-year, four-site) using a more representative natural population of 206 soybean accessions to assess genotype-by-environment interactions; (2) mine stable SCN resistance-associated genetic loci across multiple environments through GWAS based on 101,549 high-quality SNP markers obtained from high-throughput sequencing; (3) systematically elucidate the potential biological processes and metabolic pathways underlying resistance by integrating Gene Ontology (GO) and Kyoto Encyclopedia of Genes and Genomes (KEGG) enrichment analyses; and (4) focus on subcellular localization and overexpression functional validation of key candidate genes. By expanding population size, strengthening multi-environment stability validation, and deepening mechanistic insights through multi-omics data integration, this study aims to identify genetic loci with greater breeding application value, providing more reliable genetic resources and a theoretical foundation for the genetic improvement of SCN resistance in soybeans.

## 2 Materials and methods

### 2.1 Plant materials

From 2019 to 2020, 206 soybean varieties (lines) were introduced from different regions, consisting of 40 from Jilin Province, 41 from Heilongjiang Province, 37 from Liaoning Province, 10 from the Inner Mongolia Autonomous Region, 1 from Hebei Province, 1 from Beijing, 1 from Hubei Province, 1 from Shaanxi Province, 5 from the Xinjiang Uyghur Autonomous Region, 22 foreign varieties, 7 landraces, and 40 recurrent population lines (with female parent Changnong 7 ms6ms6 and male parents including Jiyu 30, Jiyu 204, Ji01556, Changnong 13, Gongjiao 96192-4, Jiyu 88, Jiyu 82, Jiunong 30, Jiyu 71, AC Hime, Jiyu 69, Jiyu 47, Heinong 41, Gongjiao H2002-25-4, Heinong 38, Hefeng 25, Suinong 28, Heihe 38, Beidou 5, Hefeng 35, Dongnong 163, Suinong 14, Hua 423, Ken 00-7184, and Mengdou 14). The male sterile lines mentioned above were developed through pedigree selection from recurrent population hybridization. Preliminary disease resistance screening for soybean cyst nematode was conducted on these materials, which were classified into different resistance categories. The harvested seeds were stored in a seed bank at 4 °C.

### 2.2 Experimental design

The experiment was conducted from 2022 to 2023 at the disease nursery of the Chifeng Academy of Agricultural and Animal Husbandry Sciences located in Xinglongzhuang Village, Chengzi Township, Songshan District, Chifeng City, Inner Mongolia Autonomous Region (118.72111°E, 42.16699°N), with an accumulated temperature≥10 °C of 2800–2900 °C, annual precipitation of 350–400 mm, and yellow loam soil; and at the disease nursery of the Chifeng Academy of Agricultural and Animal Husbandry Sciences in Xiqiao Town,Harqin Banner, Chifeng City, Inner Mongolia Autonomous Region (119.109439°E, 41.853738°N), with an accumulated temperature ≥10 C of 2900–3000 °C, annual precipitation of 380–420 mm, and yellow loam soil.

A randomized complete block design was adopted for the trial. Each material was sown manually in four rows, with a row length of 2 meters. The planting method used wide ridges with double rows, with a narrow row spacing of 40cm,a wide row spacing of 60cm, and a plant spacing of 10cm.The experiment was repeated three times, with Lee68 serving as the susceptible control. Before sowing, Stanley compound fertilizer (N-P_2_O_5_-K_2_O 18-18-18) was applied at a rate of 300 kg/ha, and timely intertillage and weeding were carried out.

Approximately 35–40 days after soybean emergence, 10 plants per material were sampled. The number of soybean cyst nematode cysts on the roots of each plant was recorded, and the average number per plant was calculated. Relevant agronomic traits were also investigated.

### 2.3 Genotyping analysis of germplasm population

Total genomic DNA was extracted from one compound leaf per plant (three plants in total) using the CTAB method, and the leaf DNA of 206 soybean germplasm resources was sequenced using specific-locus amplified fragment sequencing (SLAF-seq) technology (Li et al., 2023). High-quality single nucleotide polymorphism (SNP) sites, uniformly distributed across soybean chromosomes, were obtained via the Illumina HiSeq™2500 platform. After filtering with GATK and SAMtools, 101,549 SNP sites with a minimum allele frequency (MAF) greater than 0.05 were ultimately selected for subsequent association analysis.

### 2.4 Library construction and high-throughput sequencing

The construction and sequencing of the sample libraries were completed by Nanjing Jisihuiyuan Biotechnology Co., Ltd. Qualified genomic DNA samples were randomly fragmented into specific-sized fragments of 200–300 bp using Covaris ultrasonication (fragmentation parameters: peak incident power 175 W, duty cycle 10%, cycles per burst 200,duration 65s, processing temperature 4-7°C).This fragment size range was optimized to ensure uniform cluster generation on the Illumina sequencing platform, effective coverage of the insert fragments by paired-end reads, and compatibility with library preparation steps such as end repair and adapter ligation. The fragmented DNA was then size-selected using the Agency AMPure XP kit to enrich the sample bands within the target size range. The purified DNA samples were quantified with the Qubit dsDNA HS Assay Kit. The sheared DNA fragments underwent end repair, 3′-end A-tailing, and ligation of specific sequencing adapters. The ligation products were purified, and DNA fragments of appropriate sizes were selected. The DNA fragments were then amplified on a cBot instrument to construct the sequencing libraries. The constructed libraries were subjected to quality control, and those passing the criteria were used for sequencing. The average sequencing depth was 30× for the parental lines and 60× for the offspring. The reference genome used was the soybean genome version Wm82.a2.v1 (Song et al., 2016). SNP calling was performed using the GATK tool (McKenna et al., 2010) and variant annotation along with mutation impact prediction was conducted with the SnpEff software (Cingolani et al., 2012).

### 2.5 SNP detection and linkage disequilibrium analysis

The raw sequencing reads were filtered using Seqtk software (https://github.com/lh3/seqtk) and then aligned to the reference genome with BWA software. Subsequently, SNPs with a missing rate greater than 0.2 and a minor allele frequency (MAF) less than 0.05 were filtered out using Plink software (v1.90b6.21), resulting in 102,262 SNP loci. Linkage disequilibrium (LD) was evaluated by calculating the squared correlation coefficient (r^2^) of allele frequencies between different SNPs. The physical distance on the chromosome corresponding to the point where the r^2^ value drops to half of its maximum is defined as the LD decay distance (Zhang et al., 2017).Finally, SNP loci were converted into bed/bim/fam format files using vcftools software for population structure analysis (Danecek et al., 2011).

### 2.6 Group structure analysis and kinship analysis

A phylogenetic tree of the resource population was constructed using the Neighbor-Joining (NJ) method integrated in TASSEL v5.2.64, based on an allele frequency distance matrix. The specific parameters were set as follows: the p-distance model was used to calculate genetic distances; node support was evaluated through 1,000 bootstrap replicates; missing data were handled using pairwise deletion; and the tree topology was optimized based on the minimum evolution criterion. Finally, the topological structure and visual presentation of the phylogenetic tree were refined using the online tool iTOL (Letunic and Bork, 2019). In this study, 206 resource populations with abundant genetic variation were used for genome-wide association analysis. The population structure, linkage disequilibrium decay distance (LD decay), and phylogenetic relationships of the genome-wide association analysis population were analyzed and visualized using GAPIT v3.0 (Wang and Zhang, 2018). Population structure analysis was performed using the following two methods:1) Principal component analysis (PCA),in which principal components were computed via singular value decomposition based on genome-wide SNP data, and population stratification was visualized using two- or three-dimensional scatter plots of the first three principal components;2) the Bayesian model-based ADMIXTURE software (version 1.3.0), which inferred individual ancestry components via maximum likelihood estimation. The number of ancestral components (K) was set from 2 to 10, and 5-fold cross-validation was performed with 10 repeated runs. The optimal K value was determined based on the principle of minimizing cross-validation error. Phylogenetic relationships were visualized using the NJ tree, with branch lengths representing the degree of genetic differentiation.

### 2.7 Whole genome association analysis

Genome-wide association study (GWAS) was performed using the GAPIT R package with a mixed linear model (MLM) as the statistical model. The MLM incorporates both population structure (Q/PCA) and kinship (K) matrices, effectively controlling for population stratification and genetic relatedness among individuals, thereby reducing false-positive results. This model is the standard for GWAS of complex traits. Specifically, population structure was accounted for by including the first three principal components (PCA1-3) as fixed effects (Q matrix) in the model to control for spurious associations caused by population stratification. Genetic relatedness was controlled by including a kinship matrix (K matrix) calculated from genome-wide SNPs as a random effect in the model, which accounts for genetic correlations among individuals. A total of 102,262 high-quality SNP markers were used in this GWAS. The significance threshold was determined using the Bonferroni correction, calculated as p =0.1/n(where n = 102,262), resulting in a threshold of p=9.78×10^-5^(-log10(p)=4.01).This threshold is more lenient than the conventional p = 0.05/n, reflecting a balanced approach based on prior research experience to control the false-positive rate while minimizing the omission of potential true signals. The association between SNPs and phenotypic data was visualized using a Manhattan plot, with the Bonferroni-corrected threshold (-log10(p) = 4.01) represented as a blue horizontal line. SNP loci above this line were considered significantly associated. Additionally, a quantile-quantile (Q-Q) plot was used to assess the deviation between the observed and expected p-value distributions under the model, evaluating the overall goodness-of-fit and identifying potential biases.

### 2.8 Overexpression verification of GmC1(*Glyma.18G279900*) gene

Primers were designed using Primer Premier 6.0 software to amplify the target gene. The PCR products were detected by 1% agarose gel electrophoresis. Under ultraviolet light, the target bands were recovered using a gel extraction kit (HiPure Gel Pure DNA Mini Kit).The plasmid pART-CAM-EGFP was double-digested with XhoI and EcoRI. After digestion, the results were detected by agarose gel electrophoresis, and the linearization of the vector pART-CAM-EGFP was carried out. The target DNA fragment was mixed with the linearized vector at a molar ratio of 1:1, followed by the addition of homologous recombination enzyme. After thorough mixing of this mixture, it was incubated at a constant temperature of 37 °C for 30 minutes to facilitate the ligation of the target fragment to the vector. Subsequently, the ligation mixture was used to transform competent *Escherichia coli* cells, and positive clones were screened by colony PCR. For plasmids (pART-CAM-EGFP+GmC1) with correct sequencing results, extraction was performed using the Plasmid Mini KitI. Then, this plasmid was transformed into Agrobacterium tumefaciens GV3101.After the formation of single colonies, four single colonies were selected for each gene for colony PCR verification (Fan et al., 2024; Zhang et al., 2024).

The successfully detected Agrobacterium cultures were subjected to overnight shaking culture at 28 °C and 200 rpm. The suspended bacteria were prepared, with an OD600 value ranging from 0.2 to 1.Tobacco plants with the third and fourth leaves from the top were selected. A syringe with the needle tip removed was used to draw 1 milliliter of the suspended bacterial solution. Subsequently, the outlet end of the syringe was positioned at the predetermined transformation site on the back of the leaf. One hand was used to stabilize the front of the leaf, while the other hand slowly pressed the plunger of the syringe until the suspension evenly permeated into the leaf tissue. This method was also applied to infect other designated areas. After infection, the boundaries of the infected regions were marked. The leaf surface was lightly sprayed with water to maintain moisture and then wrapped with plastic wrap to protect the leaves from external interference. The treated tobacco plants were transferred to a culture room and kept in the dark overnight. The next day, the plastic wrap was removed. At 48 hours after injection treatment, the infected regions were excised, and their epidermis was accurately peeled off to prepare observation samples. Finally, the prepared samples were observed and analyzed in depth using Olympus laser confocal microscopy technology to observe the distribution of fluorescence signals within the epidermal cells of tobacco leaves (Zhu et al., 2024).

### 2.9 Creation of soybean materials overexpressing the GmC1 gene

Primers were designed using Primer Premier 6.0 software (**Table 1**). Using cDNA from H-10 as the template, the target gene was cloned via PCR. The PCR products were identified through 1% agarose gel electrophoresis. Subsequently, under ultraviolet light, the target bands were effectively recovered using the HiPure Gel Pure DNA Mini Kit. Next, the plasmid p5941-GFP-flag was subjected to double digestion with the two restriction endonucleases, XhoI and EcoRI. The digestion results were then verified and evaluated by agarose gel electrophoresis. The vector p5941-GFP-flag was linearized, and the target DNA fragment was mixed with the linearized vector at a molar ratio of 1:1, followed by the addition of homologous recombination enzyme. After thorough mixing, the mixture was incubated at 37 °C for 30 minutes. Subsequently, competent *Escherichia coli* cells were used for transformation, and positive clones were identified through colony PCR technology. For plasmids (p5941-GFP-flag+*GmC1*) verified by sequencing, extraction was carried out using the Plasmid Mini KitI.The plasmids were then transformed into Agrobacterium tumefaciens GV3101.After single colonies grew, four single colonies were selected for each gene for colony PCR verification (Wang et al., 2024).

**Table 1:** Primer design for *GmC1*.

Name	Sequence (5′-3′)	Tm (°C)	Amplification length (bp)
*GmC1*-p5941-GFP-flagF	ATTTGGAGAGGACACGCATGAAACTCCCCTCCGTACGA	56	1722
*GmC1*p5941-GFP-flagR	CCTTGTAGTCGGTCACTCTTGAAACAAAAGATTTCTTACTATGCATTTTCAA		

Plump,pest-and disease-free seeds of the Tianlong1 soybean variety were sterilized with 8% sodium hypochlorite in a desiccator for 16 hours. Subsequently, the sterilized seeds were placed with their hilums facing down and inoculated onto germination medium, with 20 seeds per plate. They were then incubated in the dark at 25°C for 16 hours. After germination, the seeds were removed from the germination medium. The hilum area of each seed was cut in half, the seed coats were removed, and one cotyledon with the plumule was retained while the radicle was excised. The plumule was then removed to expose the growing point, which was gently wounded with a scalpel. An Agrobacterium tumefaciens suspension with an OD600 of 0.5 was prepared and used to infect the wounded cotyledonary nodes for 4 hours, during which the conical flask was shaken five times to ensure thorough contact between the bacterial suspension and the explants. After the infection period, the infected cotyledonary nodes were placed flat on filter paper in co-cultivation medium. They were incubated in the dark at 25°C for 5 days, and co-cultivation was considered complete when punctate Agrobacterium colonies appeared on the filter paper. Well-growing cotyledonary nodes were selected, the distal ends of the hypocotyls were removed, and they were then inserted obliquely into recovery medium for 5 days. After recovery culture, the explants were transferred to shoot differentiation medium to induce shoot formation. The well-developed multiple shoots were then excised, with cotyledons and browned hypocotyls removed, and transferred to elongation medium (Deng et al., 2024). When the resistant differentiated shoots reached approximately 2cm in length, they were cut off and transferred to rooting medium for PCR detection.

## 3 Results

### 3.1 Quality distribution and base distribution of base sequencing

The Phred score (Qphred) reflects the sequencing error rate of each base, using the formula:


$$Q_{phred}=\log_{10}\left(e\right)$$

Calculated, and the Phred value is obtained through a predictive base calling error probability model during the base calling process. The corresponding relationship is shown in the **Table 2**:

**Table 2:** Correspondence table of phred value.

Phred phred score	Error rate of base calling	Correct rate of base calling	Q-sorce
10	1/10	90%	Q10
20	1/100	99%	Q20
30	1/1000	99.90%	Q30
40	1/10000	99.99%	Q40

During sequencing, the sequencing software analyzes and identifies these overlapping points through the first 4 base pairs, separating the positions of these overlapping points to ensure that each point detects a DNA molecule. Therefore, the error rate of the first few bases at the 5 ‘end of the sequencing sequence is relatively high. Meanwhile, due to the consumption of chemical reagents during the sequencing process, the sequencing error rate increases with the length of the sequenced reads. Therefore, when analyzing the quality distribution of base sequencing, the quality values of the first four bases and the last ten bases in the sample will be lower than those of the middle sequencing bases, but their quality values are all higher than Q30% (**Table 2**), indicating good sequencing quality.

**Figure 1:**
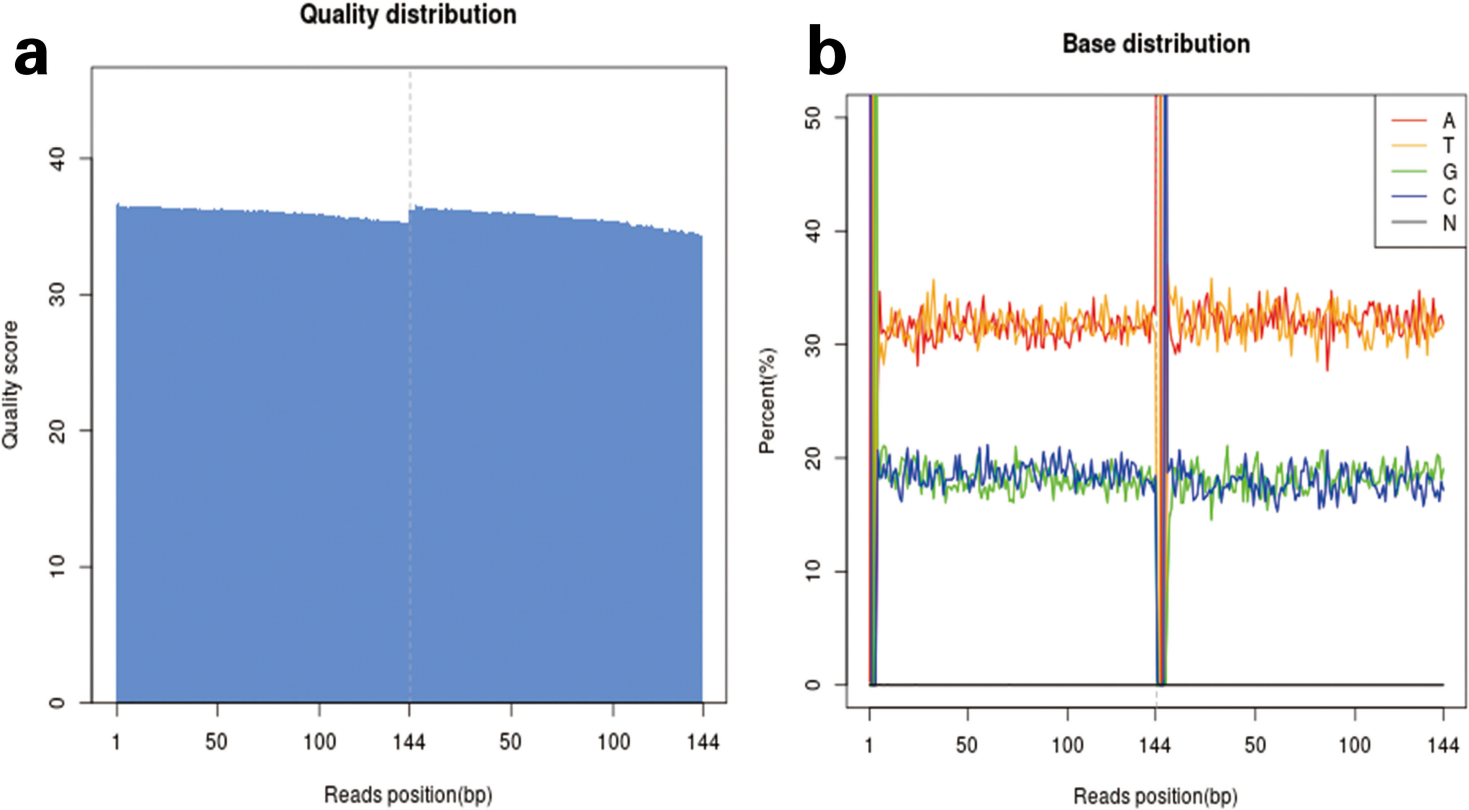
Base mass distribution and base distribution of sample.

Abnormal sequencing or library construction can lead to AT and GC separation, affecting subsequent analysis. The high-throughput measured sequence is a DNA fragment that has been randomly interrupted from the genome. The distribution of loci on the genome is approximately uniform, and the G/C and A/T content is also approximately uniform. Therefore, according to the law of large numbers, on each sequencing cycle, the GC and AT contents should be equal and equal to the GC and AT contents of the genome. Similarly, due to the overlapping clusters, the first few bases of sample R001 exhibit significant fluctuations in AT and GC, which are higher than those in other sequencing segments. However, the content of AT and CG bases GC and AT in other segments is equal and evenly distributed without separation, resulting in normal sequencing results (**Figure 1**).

### 3.2 Comparison statistics with reference genome

Based on the BWA software, sequencing data can be aligned and analyzed against the reference genome (Glycine max Wm82.a2.v1).The alignment rate (percentage of sequencing reads successfully aligned to the reference genome) and the correct alignment rate(percentage of aligned reads meeting predefined quality thresholds) are used to evaluate the degree of matching and accuracy between the data and the reference genome. By removing redundant sequences from the result files, further analyses of coverage breadth (proportion of bases in the reference genome covered by at least one read) and duplication rate (proportion of redundant sequences resulting from PCR or sequencing duplication) can be conducted. This allows for a comprehensive assessment of the coverage region and sequencing depth of the sequencing data. Partial evaluation results are shown in **Table 3** below.

**Table 3:** Statistics of mapping results of partial sequencing samples.

Sample ID	Clean reads	Mapped(%)	Properly_mapped(%)
HF1	10037980	99.05%	96.88
HF10	10676580	99.14%	95.15
HF100	12153354	99.31%	96.82
HF101	7349496	99.40%	95.45
HF102	12603738	99.42%	94.81
HF103	10450476	99.22%	96.07
HF104	12465004	99.30%	94.21
HF105	10170606	99.33%	96.47
HF106	13922716	99.34%	95.39
HF107	14398524	99.37%	94.48

The number of Clead Reads refers to two Reads (Read1 and Read2) from double ended sequencing; Comparison rate: the proportion of reads located on the reference gene; Comprehensive alignment rate: The proportion of double ended sequencing sequences that are aligned to the reference genome.

### 3.3 Distribution statistics of inserted fragments

**Figure 2:**
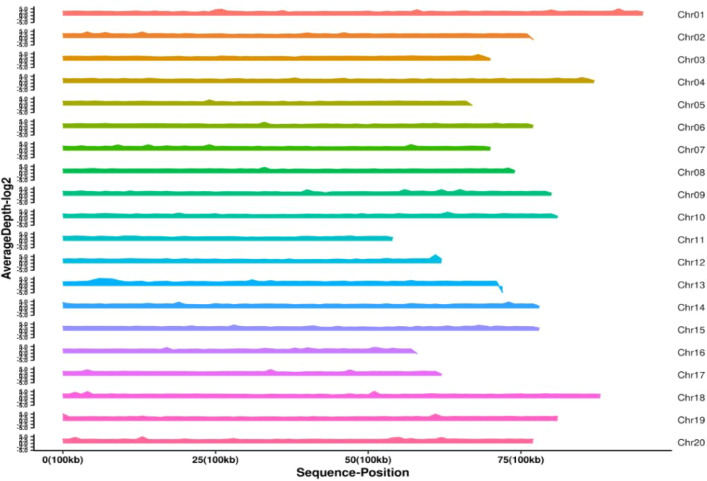
Coverage depth across chromosome of sample.

Insert Size refers to the actual size of the sequencing fragment obtained after DNA fragmentation in a sequencing sample. It is determined by detecting the start and end positions of the double ended sequence on the reference genome and is an important parameter in information analysis. The distribution of inserted fragment sizes generally follows a normal distribution and has only one single peak. Using Picard software (http://broadinstitute.github.io/picard/) The CollectInsertSizeMetric.jar software in the toolkit analyzes the length distribution of inserted fragments, and the distribution map of inserted fragments for the sample is shown in the **Figure 2**. The length distribution of inserted fragments follows a normal distribution, with a peak length of approximately 221 bp, indicating that there are no abnormalities in the construction of the sequencing data library.

Insert Size refers to the actual length of the sequencing fragment after DNA fragmentation in the sample, calculated based on the alignment positions of paired-end sequencing reads on the reference genome. It is a key parameter for evaluating the quality of library construction, and its length distribution typically exhibits a unimodal normal distribution. In this study, the distribution of insert fragments was analyzed using the CollectInsertSizeMetrics.jar tool from the Picard package. The results (**Figure 2**) showed a typical normal distribution, with a mean insert length of approximately 221 bp and a standard deviation of about 28 bp, indicating highly concentrated fragment lengths and uniform data quality. The insert size of approximately 221 bp aligns perfectly with the standard library preparation requirements for Illumina high-throughput sequencing platforms. This length falls within the optimized range of 200–300 Ipswich provides sufficient overlap for paired-end 150 bp sequencing, thereby supporting data correction and improving accuracy. Additionally, this fragment size range facilitates the formation of uniformly dense and stable sequencing clusters during bridge PCR amplification, ensuring reliable sequencing yield and base−call accuracy. In summary, both the distribution characteristics and the quantitative metrics of the insert fragments confirm that the sequencing library construction process was standardized and free of abnormalities.

### 3.4 Depth distribution statistics

**Figure 3:**
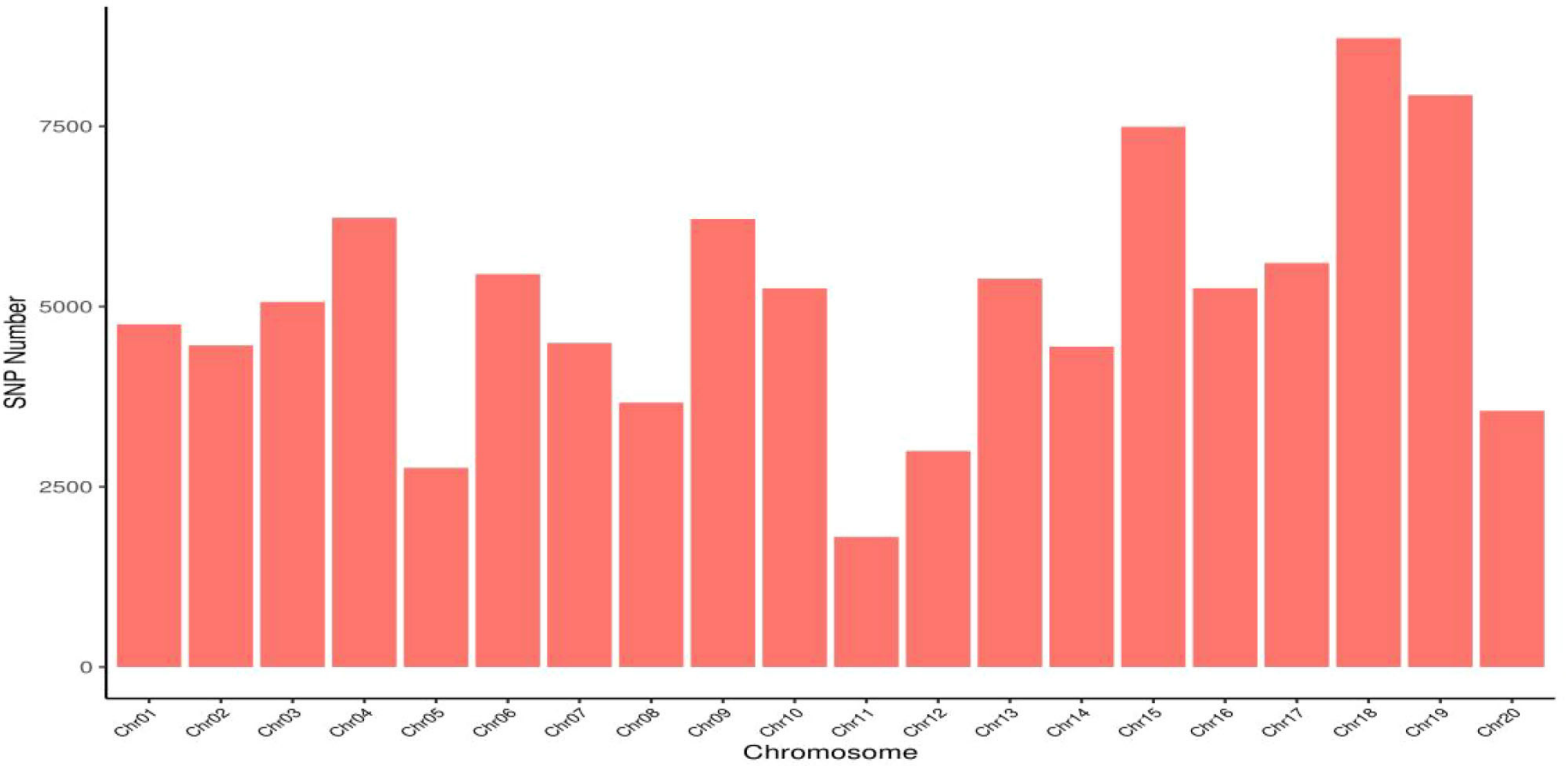
Statistical chart of chromosome SNP quantity.

Genome coverage and coverage depth are two key metrics for evaluating sequencing quality. After aligning the sequencing reads to the reference genome, the coverage of bases on the reference genome can be assessed. The percentage of bases on the reference genome covered by reads is referred to as genome coverage, while the number of reads covering a given base is defined as coverage depth. Genome coverage reflects the comprehensiveness of variant detection on the reference genome-the greater the coverage, the more variant sites can be identified. Coverage is primarily influenced by sequencing depth and the genetic relatedness between the sample and the reference genome. Genome coverage depth affects the accuracy of mutation detection; in regions with higher coverage depth (excluding repetitive sequences), mutation detection accuracy is typically higher. The genome coverage of the samples is shown in **Figure 3**, where the depth distribution of base coverage across the genome appears relatively uniform, indicating good sequencing randomness. The average coverage depth of the parental genomes exceeded 7X,with an average genome coverage above 95% (at least 1X coverage). For the offspring samples, the average coverage depth was 3X, with coverage above 80% (at least 1X coverage).The sequencing data revealed significant variation in average depth among samples. For instance, HF100 (23.39X) and HF102 (23.25X) exhibited relatively high depth, whereas samples like HF1 (9.47X) and HF105 (10.08X) showed lower depth. These differences may stem from variations in genomic complexity, DNA quality, or library construction efficiency among the germplasm resources. Despite the depth differences, the data met the basic requirements for GWAS analysis in terms of data volume. The current data demonstrated sufficient depth and good inter-sample consistency within the target regions, making it fully suitable for tag-based association analysis. The uniform distribution of coverage depth across chromosomes further confirmed good sequencing randomness and standardized library construction (**Table 4**).

**Table 4:** Coverage depth and coverage ratio statistics of partial sample.

Sample ID	Ave_depth	Cov_ratio_1X (%)	Cov_ratio_5X (%)	Cov_ratio_10X (%)
HF1	9.47	15.64%	5.27%	3.72%
HF10	7.44	21.19%	6.17%	3.82%
HF100	23.39	7.66%	4.54%	3.74%
HF101	7.39	14.66%	4.72%	3.12%
HF102	23.25	7.99%	4.54%	3.77%
HF103	12.78	12.05%	4.71%	3.59%
HF104	16.26	11.30%	4.75%	3.70%
HF105	10.08	14.88%	5.09%	3.61%
HF106	19.76	10.39%	4.78%	3.86%
HF107	19.04	11.15%	4.90%	3.92%

### 3.5 Analysis of population structure

After filtering, the SNP markers achieved uniform distribution across the genome. Among the chromosomes, chromosome 18 had the highest number of SNPs, with a total of 8,723;in contrast, chromosome 11,being the shortest chromosome, had the fewest SNPs, with only 1,804. On the 20 chromosomes, a statistical analysis was conducted using 1 Mb as a unit, revealing an average of 90.15 SNPs/Mb. Chromosome 18 exhibited the highest SNP density, reaching 154.88 SNPs/Mb, whereas chromosome 11 had the lowest SNP density, at merely 32.03 SNPs/Mb (**Figure 3**).

**Figure 4:**
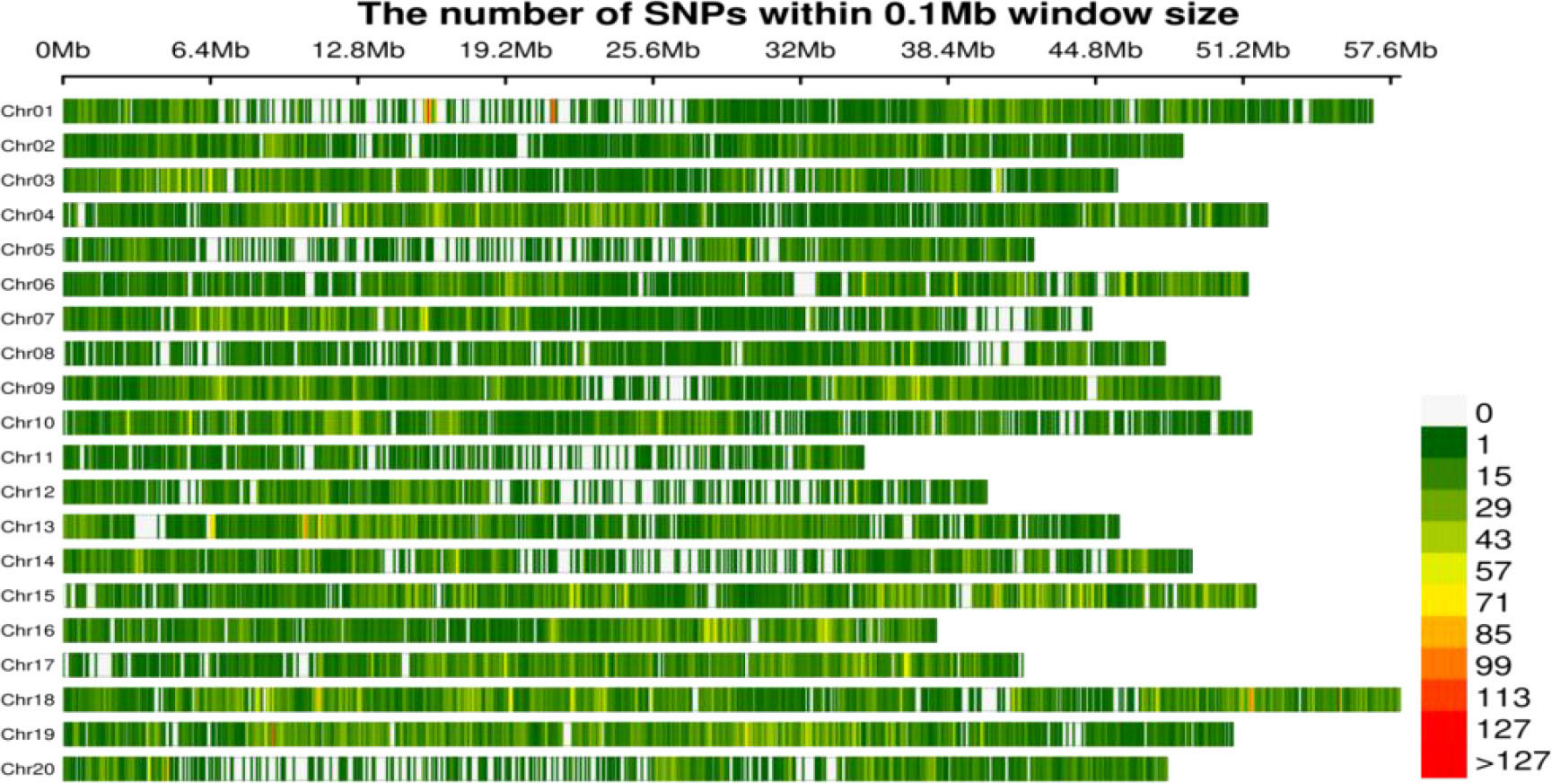
Distribution of SNP density on chromosomes.

After genotyping 206 natural populations using reduced-representation genome sequencing, a total of 101,549 high-quality single nucleotide polymorphism (SNP) markers were screened. The average sequencing depth of these markers reached 5X,and the quality score (Q) of all SNPs exceeded 30.During the filtering process, markers meeting the criteria of MQRankSum < -12.5 and ReadPosRankSum < -8.0 were retained and uniformly distributed across the 20 chromosomes. The chromosomes were divided into 100K windows, and the number of SNPs within each window was counted. A density plot was generated using the CMplot package in R (**Figure 4**).

**Figure 5:**
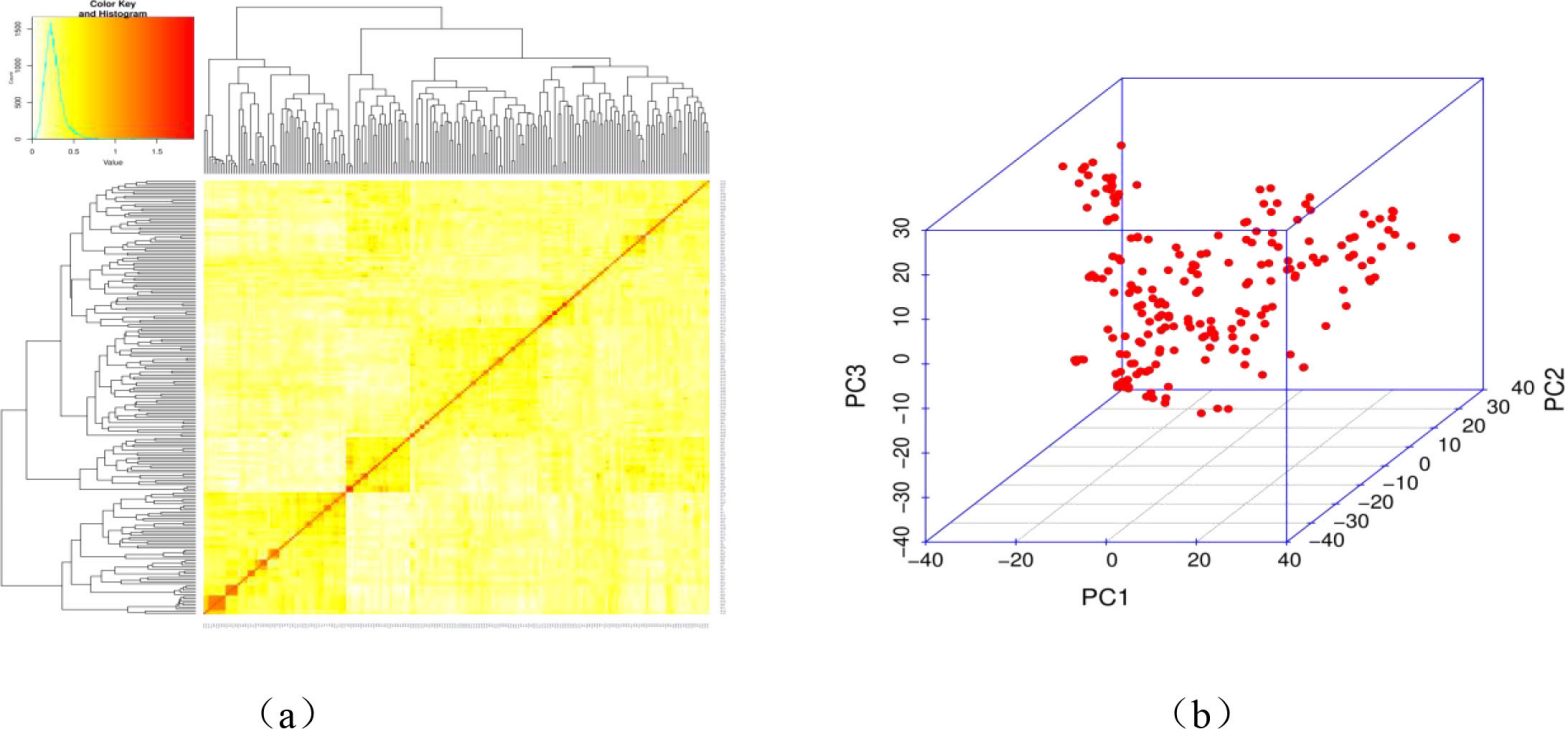
Population structure analysis.

Kinship measures the genetic similarity between two specific individuals relative to other individuals in non-familial populations or populations lacking pedigree information. When genetic relationships among individuals are imbalanced, non-linkage correlations may arise between marker loci, and the presence of small family units can increase the likelihood of false-positive results in association analyses. Therefore, in genome-wide association studies (GWAS),the kinship matrix (i.e., the K matrix) is often incorporated as a covariance matrix of random effects. In this study, the kinship matrix (K matrix) among individuals was calculated using GAPIT software and corrected during association analysis to ensure the accuracy of the results. Principal component analysis (PCA) revealed that the first three principal components cumulatively explained approximately 49.6% of the variance, with no clear clustering or stratification observed in the three-dimensional scatter plot. The kinship heatmap indicated relatively balanced kinship relationships among individuals. To correct for potential population structure effects, the first three principal components were included as covariates (combined with the K matrix correction) to ensure the reliability of the GWAS results (**Figure 5**).

### 3.6 Genome-wide association analysis for soybean cyst nematode resistance

**Figure 6:**
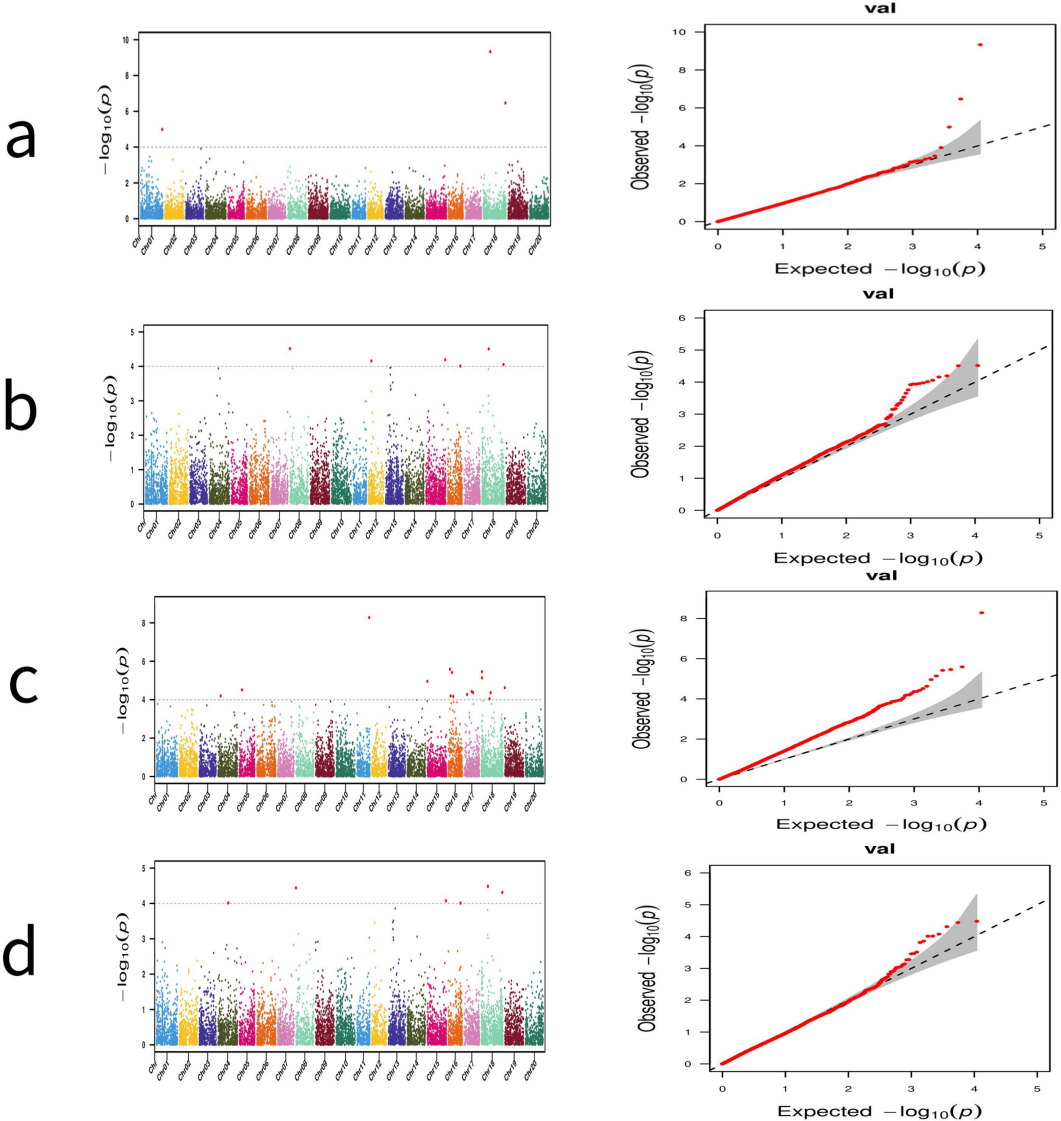
Genome wide association analysis results of soybean cyst nematode resistance. **(a)** Chengzi Base in 2022; **(b)** Xiqiao Base in 2022; **(c)** Chengzi Base in 2023; **(d)** Xiqiao Base in 2023.

Based on a natural population comprising 206 accessions, a genome-wide association study (GWAS) was conducted on the soybean cyst nematode female index (FI) under four different environments (labeled a–d) using 101,549 single nucleotide polymorphism (SNP) markers covering the whole genome and employing the mixed linear model (MLM) method. The Q–Q plot results indicated a good model fit. Although the −log10(P) values of some significant association peaks ranged between 4 and 5, which is below the strict Bonferroni-corrected threshold (P < 5×10^-8^),their significance was confirmed through permutation tests, multi−environment reproducibility validation, and model fitness assessment. Effect value analysis revealed that the locus Chr18_56072377 exhibited the strongest negative effect, indicating a significant association with enhanced resistance. A total of 19 loci significantly associated with FI were identified, which were unevenly distributed across 11 chromosomes (1, 4, 5, 8, 11, 12, 15, 16, 17, 18, 19). Specifically, 3,5,13, and 4 significant association loci were detected in environments a through d, respectively (**Figure 6**; **Table 5**). Notably, four loci (Chr18_56072377, Chr08_159841, Chr15_50312327, Chr16_32880873) were detected under multiple environmental conditions, with effect values ranging from –35.75 to 24.25. Within the candidate gene regions, we annotated several genes potentially involved in soybean cyst nematode resistance biosynthesis pathways, including but not limited to: pentatricopeptide repeat−containing protein At3g02490,proline dehydrogenase 2, isocitrate dehydrogenase, GDSL/SGNH−like acyltransferase family members, proline−rich receptor−like protein kinase PERK8, resistance proteins At4g27220 and At4g10780, phosphoribosylformylglycinamidine cyclo−ligase, E3 ubiquitin−protein ligase RGLG3, auxilin−related protein 2, serine/threonine−protein kinase RHS3, ATP−citrate synthase alpha chain protein 1,vacuolar iron transporter homolog 2,nuclear transcription factor Y subunit B−3,and VIP1 and TCP1,among others. Through comparison with previous studies, we identified Chr18_56072377 as a novel potential resistance locus, while also validating and more precisely mapping some previously reported resistance QTL regions. Based on statistical significance, multi−environment stability, and biological function, we prioritized four candidate genes, Glyma.18G279900, Glyma.18G278900, Glyma.08G001500, and Glyma.18G280300,for subsequent functional validation. Among these, the Chr18_56072377 region, owing to its outstanding multi−environment stability and the largest effect value, is considered the most promising novel major−effect resistance locus, providing an important target for molecular breeding of soybean resistance to the cyst nematode.

**Table 5:** Significant correlation loci of soybean cyst nematode female index.

Marker name	Chr.	Position	Env.	-Log10(P)	MAF	Effects	Candidate gene	Function
Chr01_55146820	1	55146820	a	3.97	0.12	19.12	*Glyma.01G222000*	mitogen-activated protein kinase homolog MMK2
							*Glyma.01G222200*	Transcription factor MYB86, MYB26
							*Glyma.01G222300*	WRKY transcription factor 22
							*Glyma.01G223000*	membrane-associated kinase regulator 2
Chr18_17560733	18	17560733	a	4.51	0.07	21.32	*Glyma.18G129200*	phospholipid-transporting ATPase 3 isoform X3
Chr18_56072377	18	56072377	a	4.06	0.5	-35.75	*Glyma.18G278400*	pentatricopeptide repeat-containing protein At3g02490
			b					
			c					
			d					
	18		a				*Glyma.18G278500*	short-chain dehydrogenase reductase ATA1
			b					
			d					
	18		a				*Glyma.18G278800*	S-adenosylmethionine decarboxylase proenzyme
			d					
	18		a				*Glyma.18G278900*	proline dehydrogenase 2, mitochondrial
			b					
			c					
			d					
	18		a				*Glyma.18G279800*	GDSL/SGNH-like acyl-esterase family protein
			b					
			c					
			d					
	18		a				*Glyma.18G279900*	GDSL/SGNH-like acyl-esterase family protein
			b					
			c					
			d					
	18		a				*Glyma.18G280200*	proline-rich receptor-like protein kinase PERK8
			b					
			d					
	18		a				*Glyma.18G280300*	disease resistance protein At4g27220
			b					
			c					
			d					
	18		a				*Glyma.18G280400*	Putative disease resistance protein At4g10780
			b					
			c					
			d					
	18		a				*Glyma.18G280700*	Transcription factor TCP1
Chr15_583863	15	583863	c	4.21	0.34	17.94	*Glyma.15G006400*	NADH dehydrogenase [ubiquinone] flavoprotein 2,mitochondrial
							*Glyma.15G007800*	G-type lectin S-receptor-like serine/threonine-protein
Chr08_159841	8	159841	b	4.52	0.07	23.32	*Glyma.08G001000*	Phosphoribosyl formylglycineamide cyclo-ligase
			d				*Glyma.08G001000*	phosphoribosyl formylglycinamidine cyclo-ligase
	8		b				*Glyma.08G001300*	nuclear transcription factor Y subunit B-3
	8		b				*Glyma.08G001500*	E3 ubiquitin-protein ligase RGLG3
			d					
	8		b				*Glyma.08G002300*	Auxilin-related protein 2
			d					
	8		b				*Glyma.08G002600*	Aldehyde dehydrogenase family 3 member I1,chloroplastic
			d					
	8		b				*Glyma.08G002700*	aldehyde dehydrogenase family 3 member I1
			d					
	8		b				*Glyma.08G003300*	CBS domain-containing protein CBSCBSPB3
Chr12_8003258	12	8003258	b	4.16	0.12	-20.9	*Glyma.12G095000*	E3 ubiquitin-protein ligase PUB24
							*Glyma.12G095200*	protein FAF-like, chloroplastic
							*Glyma.12G095300*	serine/threonine-protein phosphatase BSL3
Chr15_50312327	15	50312327	b	4.19	0.06	24.25	*Glyma.15G266500*	NAC domain-containing protein 76
	15		b				*Glyma.15G266700*	isocitrate dehydrogenase [NADP], chloroplastic
			d					
	15		b				*Glyma.15G266800*	serine/threonine-protein kinase RHS3
			d					
	15		b				*Glyma.15G267000*	ATP-citrate synthase alpha chain protein 1
			d					
	15		b				*Glyma.15G267700*	major facilitator superfamily domain-containing protein 12
			d					
Chr16_32880873	16	32880873	b	4.01	0.06	23.44	*Glyma.16G168200*	vacuolar iron transporter homolog 2-like
			d					
	16		b				*Glyma.16G168400*	transcription factor VIP1
			d					
	16		b				*Glyma.16G168500*	transcription factor VIP1-like
Chr04_7496105	4	7496105	c	4.15	0.14	30.53	*Glyma.04G087100*	serine/threonine-protein kinase Aurora-1 isoform
							*Glyma.04G087600*	Putative E3 ubiquitin-protein ligase RING1a
Chr05_7563576	5	7563576	c	4.51	0.47	33.29	*Glyma.05G070700*	serine/threonine-protein kinase BSK2
Chr11_32968092	11	32968092	c	4.74	0.08	-32.65	*Glyma.11G233800*	protein RETICULATA-RELATED 5, chloroplastic
							*Glyma.11G234800*	Transcription factor bHLH61
							*Glyma.11G235300*	CBL-interacting protein kinase 2
							*Glyma.11G235400*	Uroporphyrinogen decarboxylase, chloroplastic
							*Glyma.11G235500*	5-amino-6-(5-phospho-D-ribitylamino)uracil phosphatase
Chr17_20511505	17	20511505	c	4.43	0.06	-16.32	*Glyma.17G180400*	L-ascorbate oxidase-like isoform A
							*Glyma.17G180500*	L-ascorbate oxidase homolog
Chr16_10232275	16	10232275	c	4.35	0.53	-16.99	*Glyma.16G085700*	TMV resistance protein N isoform X1
Chr18_1729783	18	1729783	c	5.46	0.06	18.73	*Glyma.18G023200*	protein RETICULATA-RELATED 5, chloroplastic
							*Glyma.18G024000*	GDSL/SGNH-like Acyl-Esterase family
Chr16_4706786	16	4706786	c	5.59	0.11	14.4	*Glyma.16G048700*	E3 ubiquitin-protein ligase RMA2
							*Glyma.16G049000*	Sialyltransferase-like protein 1 isoform A
							*Glyma.16G049300*	aspartokinase 1, chloroplastic
							*Glyma.16G049400*	transcription factor ICE1-like
							*Glyma.16G049800*	pentatricopeptide repeat-containing protein At3g26782
Chr16_6328818	16	6328818	c	4.2	0.1	-12.51	*Glyma.16G063400*	Putative E3 ubiquitin-protein ligase RING1a
							*Glyma.16G063700*	myb-related protein 315
							*Glyma.16G064500*	LRR receptor-like serine/threonine-protein kinase ERL2
Chr17_8198521	17	8198521	c	4.87	0.19	-32.93	*Glyma.17G103400*	LRR receptor-like serine/threonine-protein kinase At1g74360
							*Glyma.17G104200*	protein ENHANCED DISEASE RESISTANCE 2-like
							*Glyma.17G105100*	Serine/threonine-protein kinase CTR1
							*Glyma.17G105500*	E3 ubiquitin-protein ligase RNF12-B
Chr18_1597524	18	1597524	c	4.23	0.26	19.32	*Glyma.18G020400*	E3 ubiquitin-protein ligase XERICO
							*Glyma.18G021300*	WEB family protein At2g40480
							*Glyma.18G021400*	5-amino-6-(5-phospho-D-ribitylamino)uracil phosphatase
							*Glyma.18G022200*	Transcription factor bHLH61
Chr19_78582	19	78582	c	4.63	0.36	8.46	*Glyma.19G000200*	Ferredoxin-thioredoxin reductase, variable chain

(a) Chengzi Base in 2022; (b) Xiqiao Base in 2022; (c) Chengzi Base in 2023; (d) Xiqiao Base in 2023.

### 3.7 GO enrichment analysis for genes at GWAS associated loci

**Figure 7:**
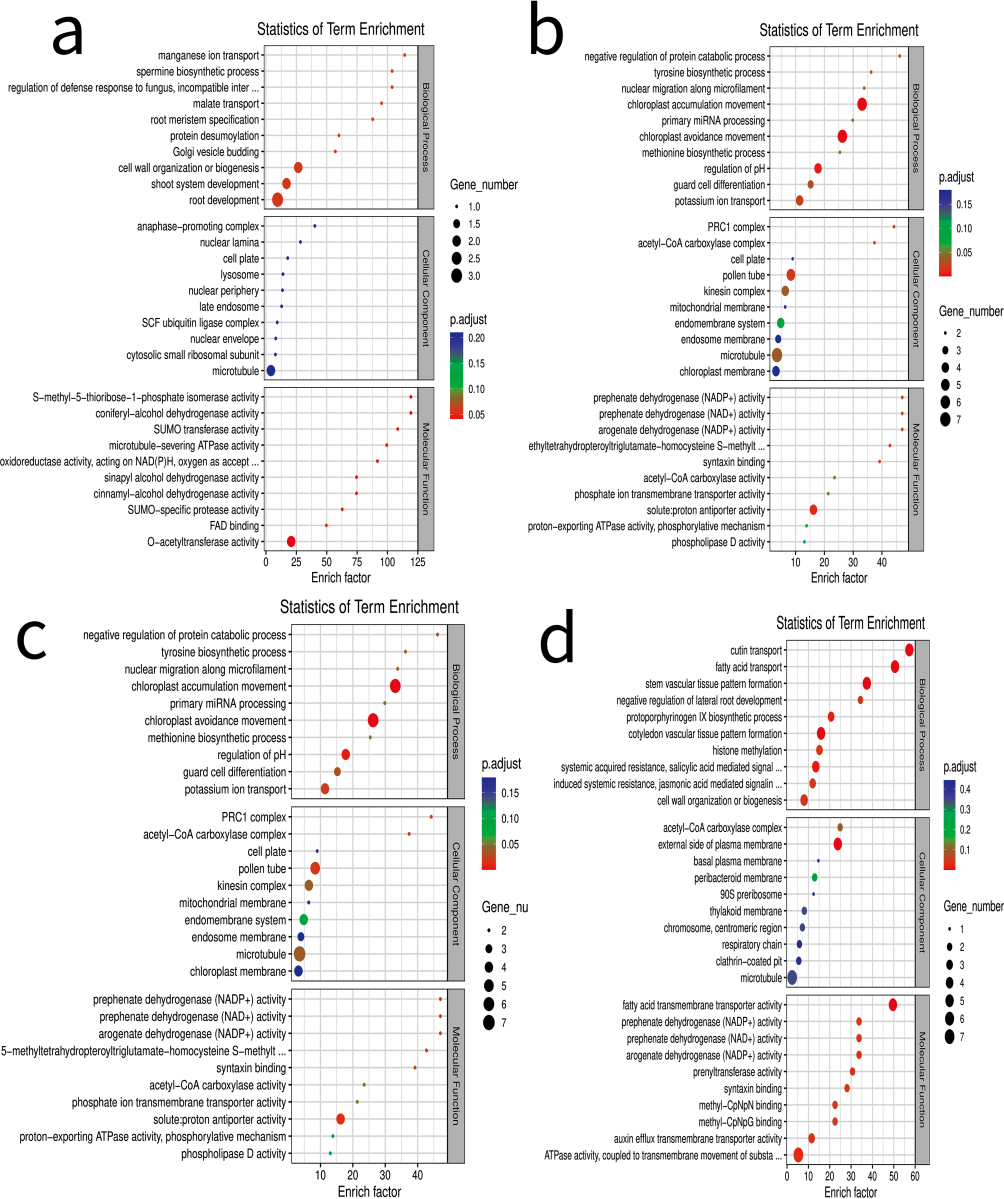
GWAS associated gene GO annotation classification. **(a)** Chengzi Base in 2022; **(b)** Xiqiao Base in 2022; **(c)** Chengzi Base in 2023; **(d)** Xiqiao Base in 2023.

GO enrichment analysis was performed on the genes associated with the four environments (**Figure 7**), with the top 10 most significant entries after FDR correction selected from each of the three categories: Biological Process (BP),Cellular Component (CC),and Molecular Function (MF).The results showed that at the Biological Process level, cell wall organization and biogenesis (GO:0010071,etc.,FDR =2.1×10^-8^), root development (GO:0048194,etc.,FDR = 5.3×10^-7^),and salicylic acid/jasmonic acid-mediated resistance signaling pathways (GO:1901332, etc. = 1.4×10^-6^/3.7×10^-6^)were significantly enriched. This indicates that soybeans defend against nematode infestation by strengthening the physical barrier of the cell wall, optimizing root architecture, and coordinately activating core defense hormone networks. At the Cellular Component level, the anaphase-promoting complex(GO:0005680, etc., FDR=6.2×10^-7^), microtubule cytoskeleton(GO:0022627, etc., FDR=1.5×10^-6^),and mitochondrial membrane (GO:0012505, FDR=4.1×10^-6^) were significantly enriched. This reveals the mechanisms by which cells enhance adaptability under stress by regulating the cell division cycle, maintaining cytoskeletal stability, and ensuring energy metabolism homeostasis. At the Molecular Function level, sulfur metabolism-related isomerases(GO:0016413, etc., FDR=8.3×10^-9^), key dehydrogenases involved in lignin synthesis(GO:0046523, etc., FDR =1.2×10^-7^),and acetyl-CoA carboxylase activity (GO:0019905, FDR=3.6×10^-7^)were significantly enriched. From a biochemical perspective, this further corroborates the central roles of antioxidant defense, enhanced cell wall lignification, and lipid metabolism remodeling in stress resistance. In summary, these significantly enriched GO terms systematically elucidate the integrated regulatory mechanisms by which soybeans respond to cyst nematode stress through a multi-dimensional synergistic network involving structural reinforcement (cell wall, roots)–signal transduction (hormone pathways)–metabolic reprogramming (sulfur metabolism, lignin synthesis, lipid metabolism).

### 3.8 KEGG enrichment analysis for genes at GWAS associated loci

**Figure 8:**
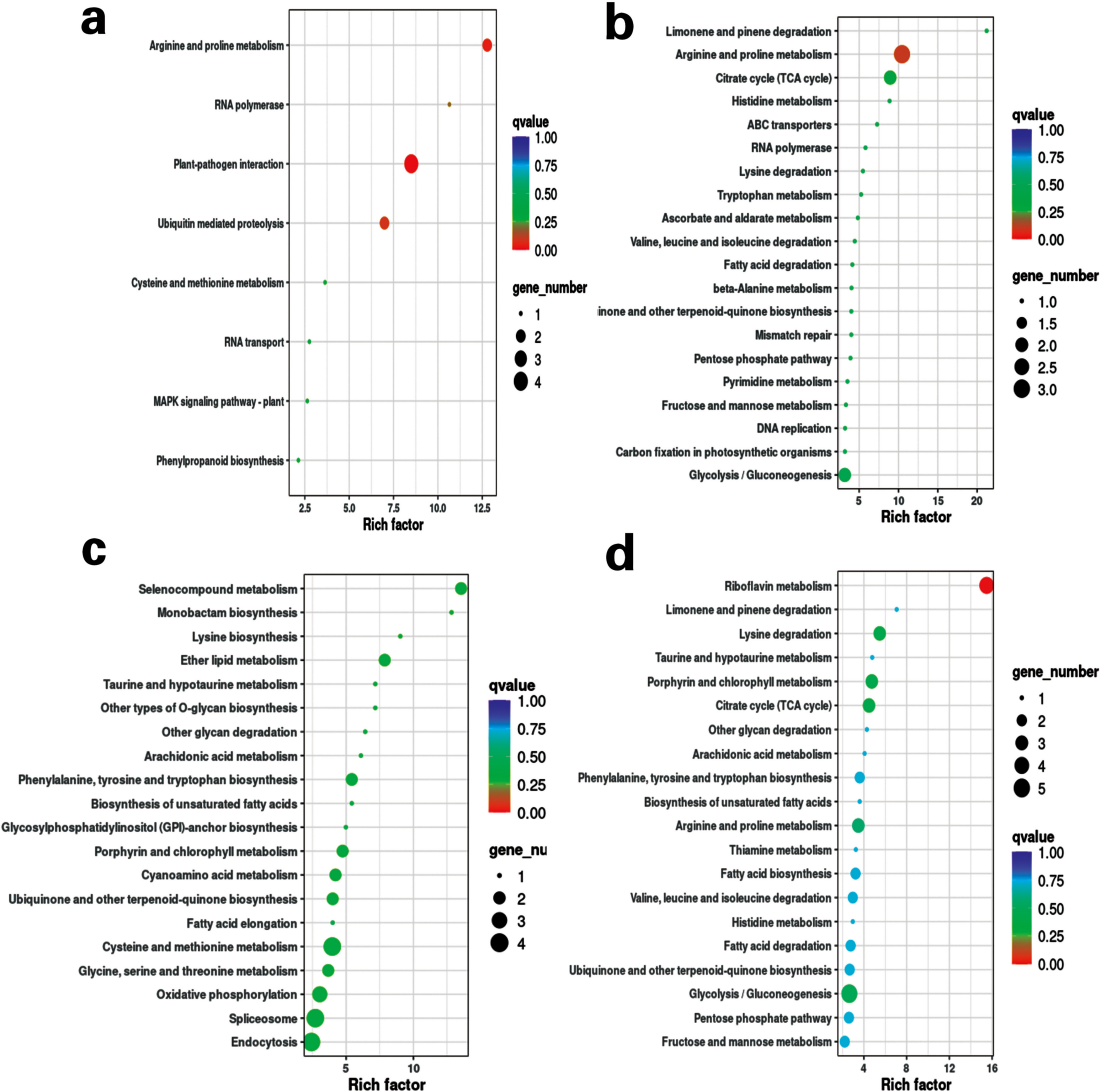
Scatter plot of KEGG pathway enrichment in GWAS associated region genes. **(a)** Chengzi Base in 2022; **(b)** Xiqiao Base in 2022; **(c)** Chengzi Base in 2023; **(d)** Xiqiao Base in 2023.

Based on the KEGG enrichment analysis results (**Figure 8**), pathway analysis was performed on genes associated with four environmental factors, selecting the top 20 core pathways with the smallest FDR-corrected P-values and highest enrichment scores. The analysis showed that Cysteine and methionine metabolism (FDR=1.2×10^-5^,enrichment score=1.8) and Biosynthesis of unsaturated fatty acids(FDR=3.4×10^-4^, enrichment score=1.5) were the most significantly enriched pathways, indicating that plants prioritize the remodeling of sulfur metabolism and membrane lipid reconstruction in response to soybean cyst nematode (SCN) infection. Additionally, the high enrichment of Glycolysis/Gluconeogenesis (FDR=5.6×10^-4^,enrichment score=1.4) and the Pentose phosphate pathway(FDR=7.8×10^-4^, enrichment score=1.3) reflects the reprogramming of energy metabolism, providing carbon sources and reducing power for defense responses. Moreover, Phenylpropanoid biosynthesis (FDR=2.1×10^-3^, enrichment score=1.2) and Ubiquinone and other terpenoid-quinone biosynthesis (FDR=4.3×10^-3^,enrichment score=1.1) were also significantly enriched, suggesting that plants reinforce cell walls and scavenge free radicals by synthesizing secondary metabolites and antioxidants.

KEGG enrichment analysis revealed that the Cysteine and methionine metabolism pathway (**Figure 8a**) was significantly enriched, promoting lignin synthesis to strengthen the cell wall barrier and driving glutathione (GSH) production to enhance antioxidant defense, thereby synergistically blocking nematode stylet penetration and neutralizing reactive oxygen species (ROS) generated during infection. The Biosynthesis of unsaturated fatty acids (**Figure 8b**) increases membrane lipid unsaturation, enhancing membrane fluidity to counteract the effects of nematode effector proteins, while also promoting the activation of the jasmonic acid (JA) signaling pathway, thereby inducing the expression of defense-related genes. The significant enrichment of the Glycolysis/Gluconeogenesis and Pentose phosphate pathways (**Figures 8c, d**) provides ATP to support cell division and defense protein synthesis, as well as essential NADPH for phenylpropanoid synthesis and the antioxidant system, forming an “energy-defense” coupled response. The Phenylpropanoid biosynthesis pathway (**Figure 8d**) produces secondary metabolites such as ferulic acid and coumarin, which inhibit nematode egg hatching and larval viability while further reinforcing the physical barrier of the cell wall, corroborating the “cell wall organization” results from the previous GO analysis. In summary, these pathways, with their significant FDR values and high enrichment scores, collectively constitute a multi-layered, systematic SCN resistance regulatory network encompassing “metabolic remodeling-energy supply-barrier reinforcement-signal activation, “comprehensively enhancing soybean defense resilience against cyst nematodes from the molecular to the cellular level.

### 3.9 Subcellular localization analysis of *GmC1*(*Glyma.18G279900*)

**Figure 9:**
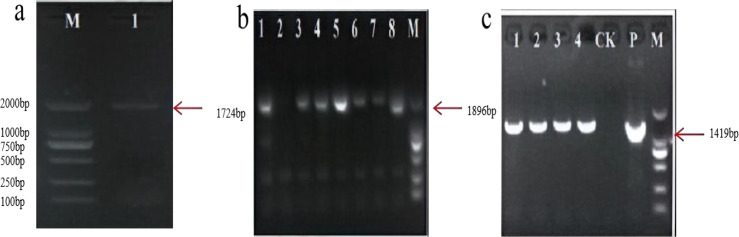
PCR amplification of *GmC1* gene. **(a)** Electrophoresis image of target gene amplification (M: Marker DL2000;I: *GmC1* gene); **(b)** Colony PCR identification of *Escherichia coli* (M: Marker DL2000;1-8:Single *E.coli* colonies with pART-CAM-EGFP+*GmC1*); **(c)** Colony PCR identification of Agrobacterium tumefaciens [M, Marker DL2000; 1-4, Single Agrobacterium colonies with *GmC1*; CK, Blank control (water); P, Positive control (plasmid)].

Based on the primers designed for the GmC1 gene in **Table 6**, the specific target band was successfully amplified by PCR (**Figure 9a**). After gel extraction and purification, homologous recombination was performed to ligate the fragment with the pART-CAM-EGFP vector. Colony PCR results following *Escherichia coli* transformation (**Figure 9b**) showed that bands of the expected size were detected in all eight single clones, indicating the successful transfer of the recombinant plasmid pART-CAM-EGFP+GmC1 into *E. coli*. Subsequently, the verified plasmids were transformed into Agrobacterium tumefaciens GV3101.Colony PCR identification (**Figure 9c**) revealed specific amplification in all four single clones and the positive control, while no band was observed in the blank control. This confirms the successful integration of the GmC1 gene into the Agrobacterium expression system, laying a foundation for subsequent genetic transformation and functional validation.

**Table 6:** Primer Design for *GmC1*.

Name	Sequence (5′-3′)	Tm (°C)	Amplification length (bp)
*GmC1*-pART-CAMEGFPF	CATTTGGAGAGGACACGCATGAAACTCCCCTCCGTACGA	56	1724
*GmC1*-pART-CAMEGFPR	TCGCCCTTGCTCACCATGAATGAAACAAAAGATTTCTTACTATGCATTTTCAA		

**Figure 10:**
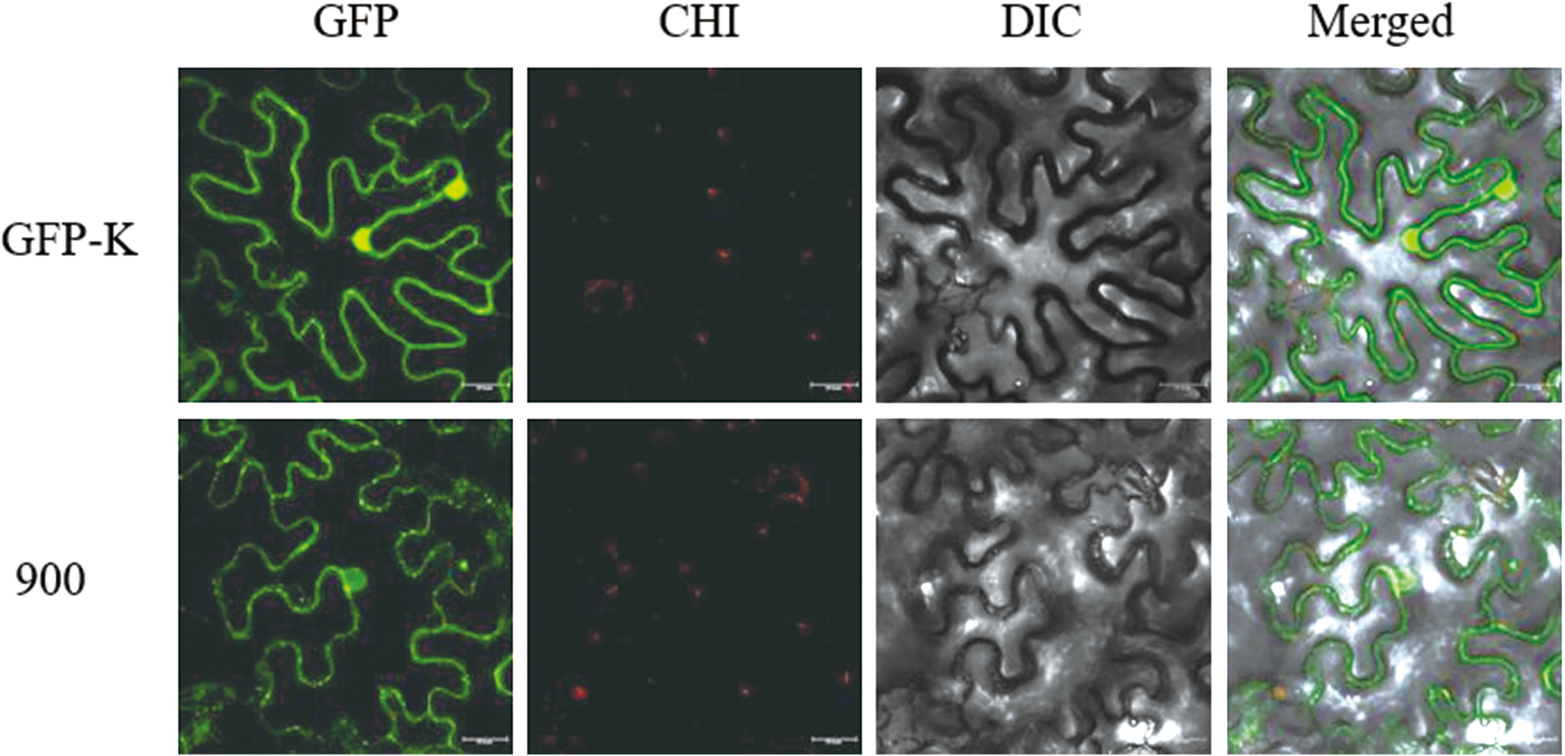
*GmC1*-GFP subcellular localization. From left to right, the images are GFP (green fluorescent protein) channel, chloroplast fluorescence channel, bright field, and overlay image, respectively.

The results showed that the *GmC1*-GFP fusion protein was significantly expressed in the nucleus, cytoplasm, and cell membrane of tobacco leaf cells (**Figure 10**).

### 3.10 Creation of soybean materials overexpressing the *GmC1* Gene

**Figure 11:**
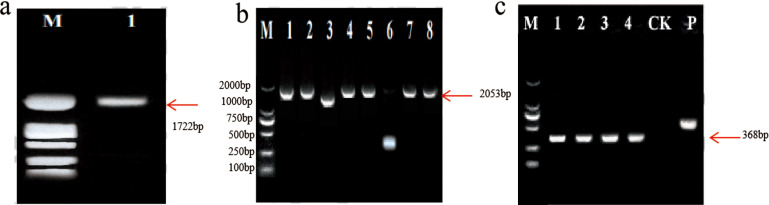
PCR amplification of *GmC1* gene. **(a)** Electrophoresis image of target gene amplification (M: Marker DL2000;1:*GmC1* gene); **(b)** Colony PCR identification of *Escherichia coli* (M:Marker DL2000;1-8:Single *E.coli* colonies with p5941-GFP-flag+*GmC1*); **(c)** Colony PCR identification of Agrobacterium tumefaciens [M, Marker DL2000; 1-4, Single Agrobacterium colonies with *GmC1*; CK, Blank control (water); P, Positive control (plasmid)].

In this study, the GmC1 gene was amplified using the primers listed in **Table 1**, and the plant expression vector p5941-GFP-flag+GmC1 was successfully constructed through a series of steps including enzyme digestion, homologous recombination, *Escherichia coli* transformation, and Agrobacterium transformation. The colony PCR results shown in **Figure 11** fully validated the accuracy and reliability of the vector construction and transformation processes, laying a solid foundation for subsequent subcellular localization and functional studies of the GmC1 gene.

**Figure 12:**
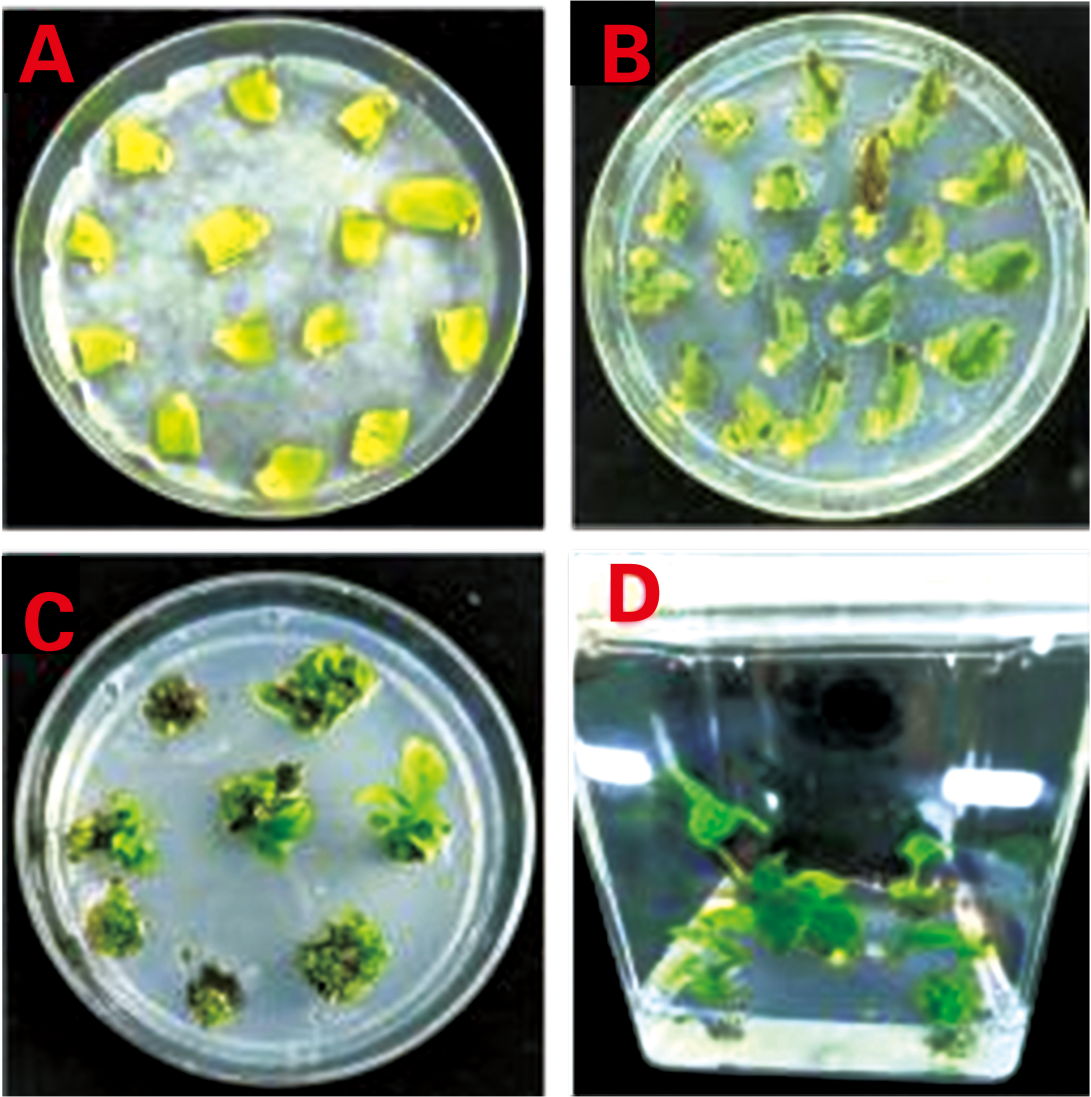
Conversion process of soybean *GmC1.* **(A)** Preparation of explants; **(B)** Soybean induction; **(C)** Generation of resistant shoots; **(D)** Rooting culture.

This procedure covers the entire process, from explant preparation, Agrobacterium-mediated infection, co-cultivation, callus induction, bud differentiation and elongation, rooting, to final molecular detection. **Figure 12** visually illustrates the complete morphogenetic process of soybean transgenic regenerated plants, demonstrating the feasibility and effectiveness of this experimental protocol in soybean genetic transformation.

### 3.11 Detection of *GmC1* transgenic soybean plants

**Figure 13:**
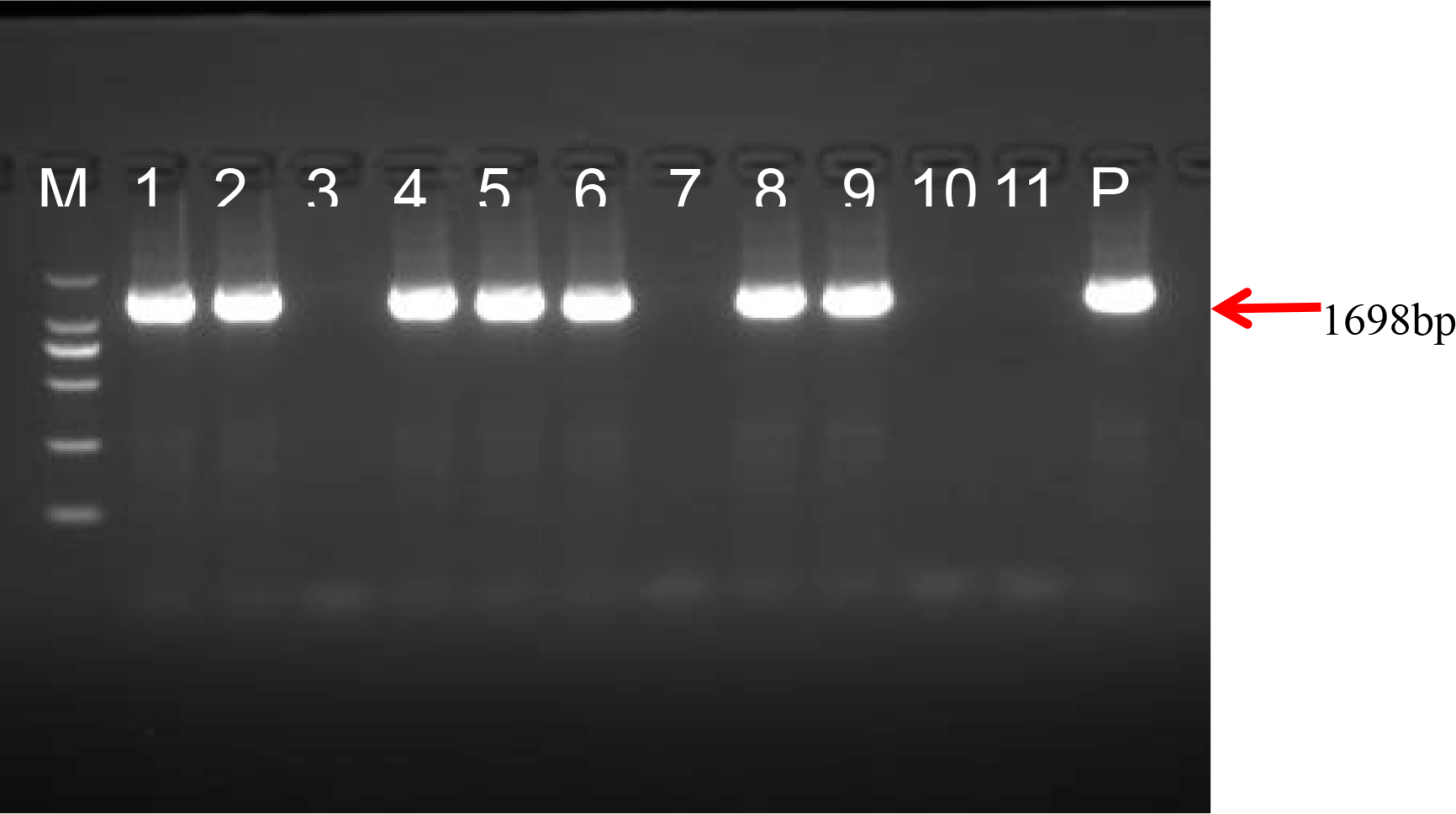
Partial plant PCR detection. [M, Marker DL2000; 1-9, T_0_ generation plants (except for plants 3 and 7, all others are positive seedlings); 10, Negative control (wild-type); 11: CK blank control (water); P, Positive control (Agrobacterium tumefaciens suspension)].

After transplanting the rooting plants, DNA was extracted from the leaves of both wild-type and transgenic soybean plants. PCR analysis was conducted to screen for 15 soybean plants with overexpression of the GmC1 gene (Liu et al., 2021). These plants were then cultivated in a controlled growth chamber, and T0 seeds were harvested, providing materials for subsequent investigations into the function of the *GmC1* gene in soybean (**Figure 13**).

### 3.12 Identification of resistance to soybean cyst nematode disease in transgenic soybean

**Figure 14:**
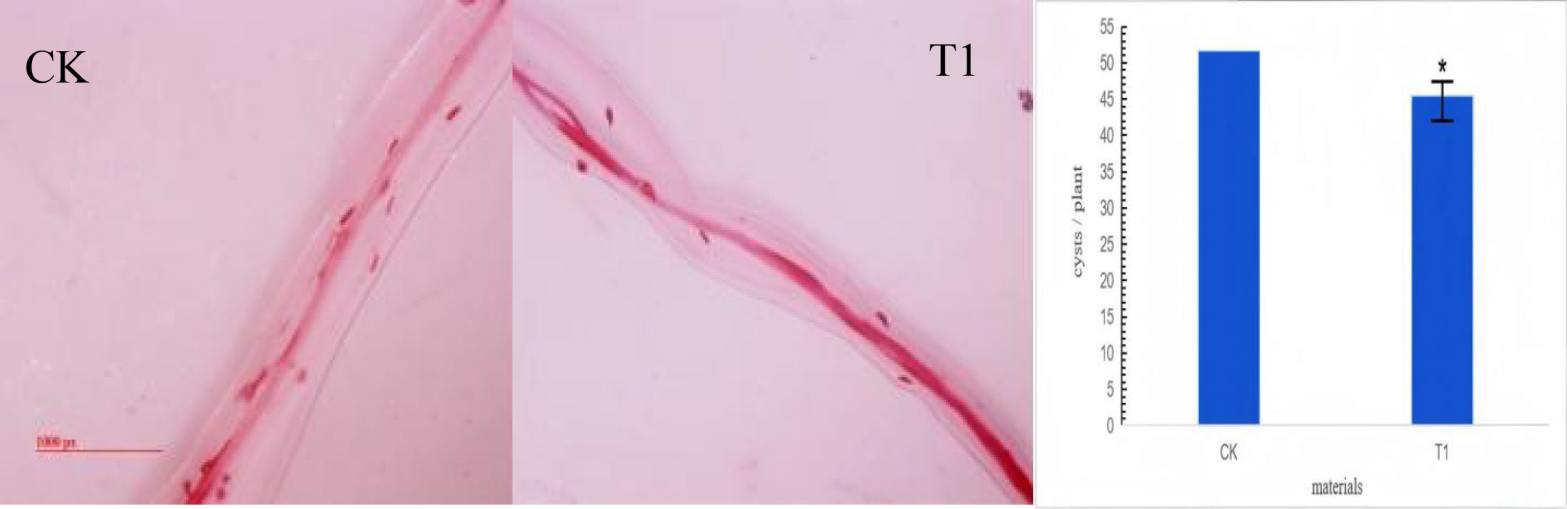
Identification of disease resistance in transgenic plants. CK, Tianlong 1 (wild-type/non-transgenic control); T1, Transgenic Tianlong 1. * and ** indicate significant correlations at the 0.05 and 0.01 levels, respectively.

The T1 generation and CK (transgenic Tianlong 1 and non-transgenic Tianlong 1) were planted in soil infested with SCN3, with 10 pots per material and three replicates. Lee68 was used as a control. Approximately 15 days after sowing, the entire plants were excavated. An improved acid fuchsin staining method was employed for staining, and the cysts were examined under a 20×optical microscope (Ren et al., 2017). The study revealed that both the T1 generation and CK exhibited high susceptibility to SCN3.The FI (Female Index) of the T1 generation was 70.54, while that of CK was 80.16, representing an 11.99% reduction compared to CK. Additionally, the number of cysts per plant decreased by 6.2. There was a significant difference in the number of nematode infections in the roots between the T1 plants overexpressing the *GmC1* gene and CK, indicating that the *GmC1* gene can enhance, to a certain extent, the resistance of soybean plants to soybean cyst nematode disease (**Figure 14**).

## 4 Discussion

This study established a cascaded analysis workflow of “BSA preliminary mapping → GWAS validation and fine mapping → transcriptome dynamic expression validation→candidate gene function prediction. “This workflow significantly improved the efficiency and reliability of candidate gene screening. The integration of transcriptomics and BSA has been successfully applied to mapping disease resistance genes. Jin (Jin et al., 2019) localized the disease resistance gene mlxbdto a 19.26 Mb candidate region on wheat chromosome 7BL using SLAF sequencing combined with BSA. Similarly, Zeng (Zeng et al., 2021) identified 12 potential genes associated with soybean pod borer stress resistance through BSA−Seq and transcriptome sequencing, including the WRKY16transcription factor, serine/threonine protein kinase, subtilisin-like protease, gibberellin, and disease resistance protein RGA3,which are novel genes conferring resistance to soybean pod borer. Shaibu (Shaibu et al., 2022) used BSA to map the genomic region associated with SCN4 resistance to chromosome 11,with GmSNAP11having a significant impact on SCN4 resistance. In this study, resistance-related regions identified by BSA were validated in natural populations via GWAS. Combined with transcriptome data under stress, five candidate genes with significant expression differences between resistant and susceptible materials and strong phenotypic associations were successfully identified.

Integrated analysis effectively overcomes the limitations of single-omics approaches: BSA−Seq enables rapid localization of major QTL intervals (Wang et al., 2022a); GWAS utilizes natural population variation to dissect genetic loci associated with phenotypes and can detect polygenic effects with minor contributions (Liu et al., 2021); transcriptomics provides dynamic expression profiles of genes under biotic stress, directly linking genomic variation to functional responses. This multi-dimensional integration allows for the systematic distinction between constitutive resistance genes and stress-induced resistance genes, and reveals their regulatory networks (Chen et al., 2020). However, integrated analysis remains constrained by the inherent limitations of each technology: BSA is sensitive to population genetic structure, GWAS is susceptible to population stratification and rare alleles, and transcriptome data only reflect expression states at specific time points, potentially missing key regulatory events. Furthermore, the integration and biological interpretation of multi-omics data impose higher demands on computational analysis and domain expertise.

The key candidate genes identified in this study establish direct associations with soybean cyst nematode resistance phenotypes:NB−LRR-type genes (Glyma.*18G280300/400*)exhibit significant synergistic effects with known resistance loci Rhg1/Rhg4 (Wang et al., 2022b). Their mechanism of recognizing nematode effector proteins to trigger ETI complements the “basic resistance” mediated by Rhg1 at the level of “specific recognition. “Recent genome-wide association studies further confirm the important role of such genes in soybean nematode resistance (Liu et al., 2023). GDSL esterase genes (Glyma.*18G279800/900*)contribute to resistance from the perspective of “structural defense” by participating in the hydrolysis of cell wall feruloyl esters, establishing physical barriers in the roots (Chen et al., 2022; Zhao et al., 2023), thereby spatially complementing the biochemical defense conferred by Rhg1.The proline dehydrogenase 2 gene (Glyma.*18G278900*),independent of major resistance genes, regulates proline−ROS metabolic homeostasis (Yang et al., 2020; Zhang et al., 2023), conferring resistance adaptability to plants under abiotic stresses such as drought, and revealing the dimension of “environmental plasticity” in resistance.

This study identified and emphasized the importance of *GmC1*. Unlike previously reported resistance genes, *GmC1* may represent a novel resistance module. Its validation work (overexpression, subcellular localization, phenotypic analysis) demonstrated its ability to effectively reduce nematode infection, though its mechanism of action likely differs from classical receptor-mediated resistance, suggesting the existence of a new resistance pathway. This provides a new resource for enriching the soybean nematode resistance gene pool. The GWAS population size in this study (206 accessions) remains insufficient for detecting minor-effect QTLs and rare alleles, potentially overlooking some important genetic variations. Future efforts should expand the population size and increase environmental replications to enhance detection power and the generalizability of the results. Regarding sequencing depth and environmental variation: the sequencing depth of BSA−Seq and RNA−seq may affect the detection of low-frequency variants and low-abundance transcripts. Additionally, while the four environments (a–d) in this study revealed genotype× environment interactions, the limited number of environments may not fully cover all conditions required for resistance expression. Analysis of the effect values of the four stable loci detected across multiple environments (e.g.,Chr18_56072377) indicates their significant contribution to resistance. However, caution is needed when interpreting their effect sizes, as GWAS-estimated effect values may be biased due to population structure,LD decay, and other factors. Comparisons with known QTLs (e.g., Rhg1) show that these new loci have moderate effect sizes but exhibit multi-environment stability, suggesting they may serve as important background resistance genes with cumulative value in breeding.

The successful cloning, vector construction, agrobacterium transformation, and preliminary phenotypic validation of GmC1 in this study confirm its potential function in nematode resistance, laying a foundation for further in-depth research. The enhanced resistance observed in overexpression materials provides direct evidence for its application prospects. However, current validation has primarily been conducted in model systems (such as hairy roots and transgenic plants),and its resistance performance under field conditions, as well as potential negative impacts on agronomic traits such as yield, remains unclear. Overexpression may also cause unintended metabolic changes, and the resistance mechanism requires detailed elucidation. The next steps involve:1) utilizing CRISPR/Cas9 to generate GmC1 knockout or knockdown mutants to verify its loss-of-function effects in a near-isogenic line background;2)conducting field evaluations of resistance and comprehensive assessments of agronomic traits.

Through the integration of multiomics technologies, this study systematically dissected the complex molecular network underlying soybean responses to cyst nematode infection. It not only validated some known resistance pathways but, more importantly, identified novel candidate genes, including GmC1,and proposed a multi layered defense model of “basic resistance (Rhg1/Rhg4)–specific recognition (NB−LRR)–physical barrier (GDSL esterase)–metabolic adaptation (ProDH2)”.This model provides a new perspective for comprehensively understanding soybean resistance to nematodes. In the future, soybean breeding for cyst nematode resistance should focus on:1)Multi−gene pyramiding breeding: Utilizing molecular marker−assisted selection or gene editing technologies to optimally pyramid major and minor effect resistance genes with different mechanisms of action (including newly discovered genes such as *GmC1*) to develop new varieties with broad spectrum, durable, and environmentally adaptable resistance.2)Balancing resistance and yield: Systematically evaluating the potential impacts of resistance genes on yield and achieving synergy between resistance and high yield through designed breeding strategies.

## 5 Conclusion

In this study, systematic disease resistance evaluation was conducted on 206 soybean accessions over a two-year period across four different environments. Combined with 101,549 high-quality SNP markers obtained from whole-genome resequencing, GWAS analysis identified 19 loci significantly associated with resistance to soybean cyst nematode. Among these, four multi-environment stable loci (Chr18_56072377, Chr08_159841, Chr15_50312327, and Chr16_32880873) were repeatedly detected across multiple environments, with effect values ranging from -35.75 to 24.25, indicating their important potential in resistance regulation. By integrating GO and KEGG databases along with functional annotations,9 key candidate genes were further screened: *Glyma.18G278400*, *Glyma.18G278900*, *Glyma.18G279800, Glyma.18G279900, Glyma.18G280300*, *Glyma.18G280400*, *Glyma.08G001500, Glyma.08G002300*, and *Glyma.16G168200*.These genes were significantly enriched in disease resistance-related pathways. Among them, overexpression studies of *Glyma.18G279900* confirmed its multi-organelle localization characteristics and its ability to significantly enhance plant resistance to soybean cyst nematode. This study is the first to systematically identify 4 environmentally stable resistance loci and nine functional candidate genes through multi-environment GWAS, providing new marker targets and genetic resources for molecular breeding of soybean cyst nematode resistance. It reveals a multi-layered synergistic resistance mechanism involving cell wall reinforcement, reactive oxygen species metabolism regulation, and immune signal transduction. The findings offer precise targets for molecular marker-assisted selection and gene-editing breeding, demonstrating direct breeding application value. Furthermore, the established systematic research framework of “multi-environment phenotypic evaluation GWAS mapping-multiomics integration gene functional validation” provides a referable technical pathway for the genetic dissection of complex resistance traits in crops.

## Data Availability

Publicly available datasets were analyzed in this study. This data can be found here: NCBI : PRJNA1149502.
