# Mining resistance genes of soybean cyst nematode based on genome-wide association study

**DOI:** 10.3389/fpls.2026.1910564

**Published:** 2026-06-29

**Authors:** Haibo Hu, Zhihui Cui, Depeng Wu, Qingyan Zhang, Xiaolei Wang, Yingchun Liu, Zeran Kang, Xue Wei, Liuxi Yi, Yang Wu, Huimin Shi, Yunshan Wei, Xuechao Zhou

**Affiliations:** 1 Chifeng Academy of Agricultural and Animal Husbandry Sciences, Chifeng, Inner Mongolia, China; 2 Collaborative Innovation Center of Regional Modern Agriculture & Environmental Protection Coconstructed by the Province and Ministry, Huaiyin Normal University, Huai’an, China; 3 Chifeng Meteorological Bureau, Chifeng, Inner Mongolia, China; 4 College of Agriculture, Inner Mongolia Agricultural University, Hohhot, Inner Mongolia, China

**Keywords:** candidate gene, genome wide association study, overexpressing plants, single nucleotide polymorphism, soybean cyst nematode

An incorrect **Funding** statement was provided.

“The author(s) declared that financial support was received for this work and/or its publication. Funded by “Biological Breeding-National Science and Technology Major Project”(2023ZD04032); Supported by the earmarked fund for CARS-04-CES11(CARS-04-CES11); National Key R&D Project: Research and Integrated Demonstration of Full-Mechanization Technology for Removing Obstacles, Increasing Yield, and Enhancing Efficiency in Soybean Production (2024YFD2300103); Joint Breeding Research Project of Inner Mongolia Autonomous Region: “Germplasm Innovation and New Variety Breeding of High-yielding and High-quality Soybean” (YZ2023005); Supported by the earmarked fund for CARS-04-CES11(CARS-04-CES11); Cultivation Project for a New-type R&D Platform in the Modern Agriculture Industrial Park of Horqin Right Front Banner, Inner Mongolia Autonomous Region (2026XXYF03).”

The correct **Funding** statement is:

“The author(s) declared that financial support was received for this work and/or its publication. Joint Breeding Research Project of Inner Mongolia Autonomous Region: “Germplasm Innovation and New Variety Breeding of High-yielding and High-quality Soybean” (YZ2023005), Biological Breeding-National Science and Technology Major Project”(2023ZD04032), National Key R&D Project: Research and Integrated Demonstration of Full-Mechanization Technology for Removing Obstacles, Increasing Yield, and Enhancing Efficiency in Soybean Production(2024YFD2300103), Supported by the earmarked fund for CARS-04-CES11(CARS-04-CES11), Cultivation Project for a New-type R&D Platform in the Modern Agriculture Industrial Park of Horqin Right Front Banner, Inner Mongolia Autonomous Region (2026XXYF03)].”

The original version of this article has been updated.

